# Wastewater Treatment with Bacterial Representatives of the *Thiothrix* Morphotype

**DOI:** 10.3390/ijms25169093

**Published:** 2024-08-22

**Authors:** Maria V. Gureeva, Maria S. Muntyan, Nikolai V. Ravin, Margarita Yu. Grabovich

**Affiliations:** 1 Department of Biochemistry and Cell Physiology, Voronezh State University, Universitetskaya pl., 1, 394018 Voronezh, Russia; margarita_grabov@mail.ru; 2 Belozersky Institute of Physico-Chemical Biology, Lomonosov Moscow State University, Leninskie Gory, 119991 Moscow, Russia; 3 Institute of Bioengineering, Research Center of Biotechnology of the Russian Academy of Sciences, Leninsky Prospect, 33-2, 119071 Moscow, Russia; nravin@mail.ru

**Keywords:** *Thiothrix*, *Thiolinea*, *Thiofilum*, wastewater

## Abstract

Bacteria of the *Thiothrix* morphotype, comprising the genera *Thiothrix*, *Thiolinea* and *Thiofilum*, are frequently encountered in domestic and industrial wastewater treatment systems, but they are usually not clearly differentiated due to the marked similarity in their morphologies. Methods ranging from light microscopy, FISH and PCR to modern high-throughput sequencing are used to identify them. The development of these bacteria in wastewater treatment systems has both advantages and disadvantages. On the one hand, the explosive growth of these bacteria can lead to activated sludge bulking or clogging of the treatment system’s membranes, with a consequent decrease in the water treatment efficiency. On the other hand, members of the *Thiothrix* morphotype can improve the quality of granular sludge and increase the water treatment efficiency. This may be due to their capacity for sulfide oxidation, denitrification combined with the oxidation of reduced sulfur compounds, enhanced biological phosphate removal and possibly denitrifying phosphate removal. The recently obtained pangenome of the genus *Thiothrix* allows the explanation, at the genomic level, of the experimental results of various studies. Moreover, this review summarizes the data on the factors affecting the proliferation of representatives of the *Thiothrix* morphotype.

## 1. Introduction

Representatives of filamentous bacteria, similar in morphotype to *Thiothrix*, are attaching filamentous organisms, many of which are capable of forming rosettes, accumulating elemental sulfur inside cells and spreading by gonidia [1]. In running water containing hydrogen sulfide, they form abundant whitish filamentous fouling, visible to the naked eye. In the presence of reduced sulfur compounds, representatives of the *Thiothrix* morphotype are known for their versatile metabolism, which gives them potential for lithoautotrophic and lithoheterotrophic growth [2,3,4,5]. Most commonly, they can be found in sulfide springs, deep-sea hydrotherms, irrigation systems and activated sludge from sewage treatment plants [2,3,4,5].

Over the last two decades, the taxonomy of the genus *Thiothrix* has been revised several times. In the guidelines for the identification of microorganisms that cause the bulking of activated sludge, filamentous bacteria similar in morphotype to *Thiothrix* were classified as Type 021N, *Thiothrix* I and *Thiothrix* II [6,7]. However, it is worth noting that, even at the time of the publication of these manuals, such classification was shown to be phylogenetically incorrect [8,9]. Subsequently, Boden and Scott [10] proposed that *Thiothrix disciformis* and *Thiothrix eikelboomii*, previously included in Type 021N, should be classified as the genus *Thiolinea*, while *Thiothrix flexilis* should be classified as the genus *Thiofilum*.

Despite the fact that *Thiolinea* and *Thiofilum* were assigned to separate genera 6 years ago, representatives of these genera are often still attributed to the genus *Thiothrix* in works on biological wastewater treatment [11,12]. Moreover, even the more obsolete name Type 021N was used to refer to them for nearly two decades [13,14,15,16,17,18].

**Figure 1:**
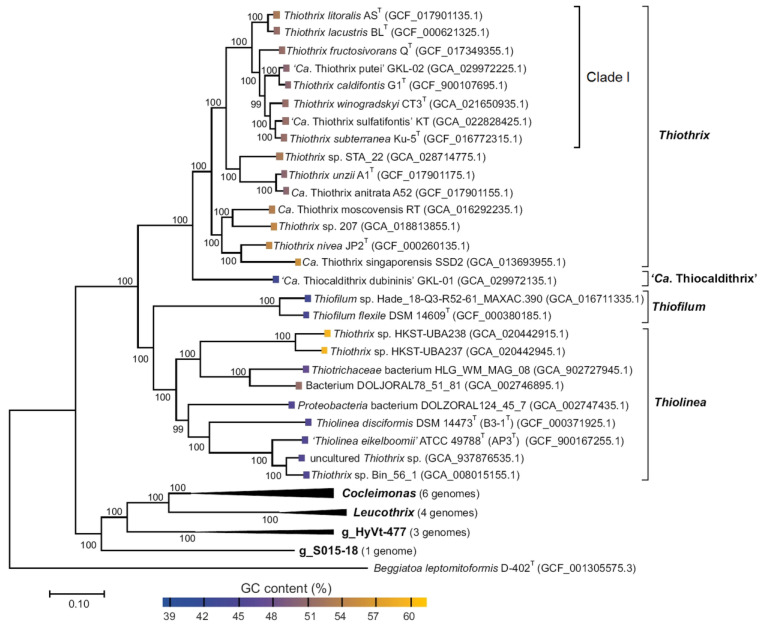
Phylogenetic tree of the family *Thiotrichaceae* based on the concatenated genomic sequences of 120 conserved marker genes (maximum likelihood method). Clade I of the genus *Thiothrix* is marked with a square bracket in the right part of the figure. For tree rooting, the genome of *Beggiatoa leptomitoformis* D-402^T^ (GCF_001305575.3) was used. Figure was modified from Ravin et al. [19] under the terms of the Creative Commons Attribution 4.0 International License.

To eliminate such confusion in taxonomy, in the presented review, we combine data on bacteria currently assigned to three genera, *Thiothrix*, *Thiofilum* and *Thiolinea*, based on phylogenetic data, in contrast to their previous assignment to the genus *Thiothrix* only on the basis of the morphotype (Figure 1).

The importance of bacteria of the *Thiothrix* morphotype in applied studies is due to the fact that they are often found in domestic [18,20,21,22,23] and industrial [24,25,26] wastewater treatment systems. Biological treatment using complex microbial communities to degrade pollutants into low-toxicity or non-toxic products is the most important method for wastewater treatment [27]. Bacteria of *Thiothrix* morphotype are often found in such microbial communities. These bacteria have been detected in wastewater treatment plants in the pulp and paper [28,29,30,31] and textile industries [32], as well as in wastewater from bisphenol A production [33]. In addition, bacteria of the *Thiothrix* morphotype actively proliferate in wastewater from food industries, including the dairy [34], fish canning [35] and potato industries [36].

Bacteria of the *Thiothrix* morphotype are often found in wastewater from pharmaceutical plants. It was shown that *Thiothrix* was one of the dominant populations at the genus level in a decomposition reactor for the wastewater treatment of lincomycin [37], diclofenac and clofibrin acid [23]; spiramycin, oxytetracycline and streptomycin [38]; and tetracycline and sulfamethoxazole [39,40].

The role of bacteria with a morphotype similar to *Thiothrix* in wastewater treatment systems is controversial. On the one hand, a certain change in the environmental parameters can cause their explosive growth, leading to activated sludge bulking and the clogging of membranes, which significantly reduces the efficiency of treatment [26,29,41]. Bulking can be related to the ability of *Thiothrix*-like organisms to form flocks with poor settling characteristics [42], which raises operational problems in biological wastewater treatment systems worldwide [18,25]. At the same time, a number of studies show that, under properly selected conditions, representatives of the *Thiothrix* morphotype, on the contrary, can help to improve the quality of granular sludge and increase the efficiency of wastewater treatment [43,44].

In addition to affecting the characteristics of activated sludge, bacteria of the *Thiothrix* morphotype are capable of performing a number of metabolic processes leading to the removal of pollutants from wastewater. These include sulfide oxidation [45], denitrification coupled with the oxidation of reduced sulfur compounds [23,46], the enhanced biological removal of phosphorus [47] and possibly the denitrifying removal of phosphorus [48,49].

Although the role of *Thiothrix*, *Thiofilum* and *Thiolinea* in the operation of wastewater treatment plants is well known and undisputed, no review has yet been published that discusses the role of these particular bacteria in wastewater treatment systems, rather than filamentous bacteria in general. The aim of this review is to summarize the current information on the types of wastewater treatment plants in which representatives of these three genera occur, the methods for their detection in wastewater and the metabolic processes that they carry out in wastewater treatment, as well as the conditions affecting the development of this group of bacteria. This review is the first to attempt to delineate the available published data on members of the *Thiothrix* morphotype into three genera, *Thiothrix*, *Thiofilum* and *Thiolinea*, according to recent advances in molecular phylogeny. It is also the first review to discuss information on the metabolic processes occurring in members of the genus *Thiothrix* in wastewater treatment plants in the context of its pangenomic data.

## 2. Types of Wastewater Treatment Plants Where *Thiothrix* Morphotype Can Occur

Microorganisms can be used in biological wastewater treatment in attached form or in suspension form [50]. In the first case, the microorganisms attach to a rigid surface on which they form a biofilm, such as in a rotating biological contactor (RBC); in the second case, the microorganisms are in suspension, as in the conventional activated sludge (CAS) process and in the membrane bioreactor (MBR) [51].

### 2.1. Activated Sludge

Activated sludge is a community of microorganisms contributing to the biological treatment of wastewater. Although filamentous bacteria are usually present in activated sludge in relatively low numbers, they can cause negative phenomena. Under certain conditions, they proliferate to such an extent that they significantly affect the performance of the wastewater treatment plant, causing sludge bulking and foaming [35]. Wastewater treatment plants in the food [34,35,36] and pulp and paper industries [28,29] face similar problems.

Most filamentous bacteria in activated sludge belong to the *Thiothrix* morphotype [52]. An analysis of the bacterial populations of 29 industrial wastewater treatment plants using 16S rRNA amplicon sequencing showed that activated sludge bulking was associated with the *Thiothrix* morphotype in 72% of cases [24].

Pure cultures belonging to the genus *Thiothrix* were repeatedly isolated from activated sludge samples [53,54]. Some isolated strains were subsequently described as representatives of new species and genera: *Thiothrix eikelboomii* (later reclassified as a member of the genus *Thiolinea* [10]), *Thiothrix unzii*, *Thiothrix fructosivorans* [8], *Thiothrix disciformis* [9] = *Thiolinea disciformis* [10] and *Thiothrix flexilis* [9] = *Thiofilum flexile* [10]. Based on the genome obtained from the metagenome (MAG) of activated sludge, a new species, *Thiothrix winogradskyi*, was described [55].

Besides pure cultures, new species of *Thiothrix* with *Candidatus* status were described in activated sludge. In this way, ‘C*andidatus* Thiothrix moskovensis’ and ‘*Ca.* Thiothrix singaporensis’ were described based on metagenome-assembled genomes (MAG) obtained from laboratory-scale bioreactors with the enhanced biological removal of phosphorus [56].

### 2.2. Aerobic Granular Sludge

As a promising alternative to the wastewater treatment process involving activated sludge, aerobic granular sludge (AGS) has received increasing attention in recent decades. In contrast to flocculated activated sludge, AGS is a granular microbial aggregate with a spatially layered structure (anaerobic/microaerobic/aerobic from inside to outside) that promotes the growth of microorganisms with diverse functionalities in one small unit [57].

In treating textile wastewater, AGS was shown to be more effective than conventional activated sludge with *Thiothrix* filaments disappearing from AGS after such treatment, resulting in improved sludge settleability [32]. However, it should be noted that AGS can potentially also face bulking problems due to the proliferation of bacteria similar in morphotype to *Thiothrix*. At the same time, it was shown that in the presence of bacteria of the *Thiothrix* morphotype (51.4 ± 8.3% of the total microbial population), stable aerobic granular sludge can be formed, and the morphology of the granules is largely determined by the operation of the reactor and, to a much lesser extent, by the morphologies of individual cells [44]. Moreover, members of the *Thiothrix* morphotype can be among the dominant bacteria in filamentous granular sludge (FGS), which is capable of maintaining relatively stable carbon and phosphorus removal efficiency but is challenging in terms of nitrogen removal [43].

An important factor affecting the granulation and sedimentability of AGS is the food-to-mass ratio (F/M) [58]. As this ratio increases in the first stages of wastewater treatment, the number of bacteria of the *Thiothrix* morphotype increases significantly, which decreases the efficiency of AGS precipitation. However, the gel-like polysaccharides associated with these bacteria effectively help to preserve the granular biomass, which gradually leads to a decrease in the F/M and the restoration of AGS’ sedimentability [58]. The C/N ratio also has an important effect on the characteristics of activated sludge and the development of *Thiothrix*. A decrease in this ratio led to increased competition between different microorganisms, including the dominant bacteria from the genus *Defluviicoccus* and the *Thiothrix* morphotype, which reduced the efficiency of the system [59]. Nutrient deficiency, especially nitrogen, also had a negative effect on AGS systems, causing sludge bulking due to the proliferation of the *Thiothrix* morphotype [60].

Incorrectly selected conditions often lead to a significant increase in the size of the granules due to an increase in the number of bacteria similar in morphotype to *Thiothrix* and the disruption of the system [61].

### 2.3. Membrane Bioreactors

Membrane bioreactors (MBRs) are modern, high-intensity biological treatment facilities. In membrane bioreactors, the separation of activated sludge flocks from treated wastewater is achieved by filtering the sludge mixture through an ultrafiltration or microfiltration membrane, after which microorganisms are removed from the membrane surface by an aeration system [62].

Challenges in operating membrane bioreactors are related to frequent membrane clogging, including the growth of filamentous bacteria [63]. *Thiothrix eikelboomii* (= *Thiolinea eikelboomii*) was reported to decrease the sludge sedimentability, increase the apparent viscosity in aerobic environments and cause dense biofilm formation on the membrane surface [12]. Membrane contamination due to an increase in the number of bacteria of the *Thiothrix* morphotype is more active at low temperatures [41].

Several methods have been proposed to offset the negative effects of bacteria of the *Thiothrix* morphotype in MBR operation. The overgrowth of *Thiothrix*-like organisms was suppressed by using micellial granules to granulate activated sludge [64], by the use of high sludge concentrations in the MBR [63] and by backwashing the membrane with NaClO [65].

### 2.4. Latest Inventions for Membrane Reactors

It should be especially noted that, for organic pollutant removal, which is difficult when using traditional biodegradation methods, chemical methods with the application of strong oxidizing agents, such as peroxymonosulfate, hydrogen peroxide, etc., have long been used as an alternative. To enhance the action of oxidizing agents, catalysts based on perovskite oxides are used. However, with the growth of industry in recent years, the insufficiency of this variant of the method has been noted, which has stimulated a search for new solutions to improve the efficiency of wastewater treatment.

For this purpose, the latest layered perovskite oxides of the Ruddlesden-Popper structure have recently been synthesized and shown to serve as an effective next-generation catalyst for the activation of peroxymonosulfate, an oxidant used in the oxidative removal of organic pollutants in modern wastewater treatment plants. Unlike conventional perovskites, in Ruddlesden-Popper perovskites, the catalytic role in the decomposition of the pollutant chemical compound is played not by free radical forms of oxygen but by non-free singlet oxygen radicals. For this reason, the newest Ruddlesden-Popper perovskite oxides have demonstrated exceptional efficiency in phenol removal (complete removal from wastewater in 10 min) [66].

The development of new Ruddlesden-Popper perovskite oxides in 2023 showed that they can serve as an effective catalytic component in next-generation composite membranes, increasing the speed and safety of the organic wastewater treatment process. In contrast to conventional perovskite oxides with the formula ABO_3_, the Ruddlesden-Popper perovskite oxides of the A_2_BO_4_ type have improved catalytic properties.

In search of materials with optimal properties, Yang et al. [67] synthesized a La_2_CoO_4_-δ catalyst with a Ruddlesden-Popper structure, in one of the best variants of which the A-site was 50% replaced by strontium to obtain the LaSrCoO_4_-δ structure. The use of such a catalyst allowed the complete oxidative decomposition of tetracycline hypochloride in 14 min. The obtained compound embedded in the matrix of a polyacrylonitrile ultrafiltration membrane gave a composite material of extraordinary efficiency in wastewater treatment, the use of which resulted in the 94% degradation of tetracycline hypochloride in only 3 min and a significant reduction in the wastewater’s toxicity.

### 2.5. Biofilms

A biofilm is a set of microorganisms located on the surface of wastewater or parts of treatment facilities. One of the most common types of bioreactors that contain microorganisms in the form of a biofilm is the rotating biological contactor (RBC).

In an RBC, a chain of round plastic discs rotates on a horizontal rod using a motor. This rotation causes the mixing of wastewater volumes, the diffusion of compounds into the biofilm, convection through the pores/medium of the biofilm and the subsequent exchange of products with the reactor and the environment. Oxygen diffusion is obligatory for the oxidation of organic substances. The rotation of the discs creates shear forces that inhibit excess biofilm growth. Excess sludge is transferred to a settling tank [68].

Filamentous organisms can proliferate at the surface–biofilm interface in RBCs. The presence of filaments of the *Thiothrix* morphotype in RBC biofilms has been shown many times, although they are much less common in biofilms than in suspension form. They were detected as part of an RBC biofilm in the simultaneous removal of nitrogen and phosphorus [21]. It was shown that the *Thiothrix* morphotype can be used as an indicator of nitrous and nitric nitrogen’s presence in RBCs [69]. In addition, bacteria similar in morphotype to *Thiothrix* were found in the biofilm of a non-aerated reactor for the treatment of sulfide-containing wastewater [70].

## 3. Methods for Identification of *Thiothrix* in Wastewater Treatment Plants

**Figure 2:**
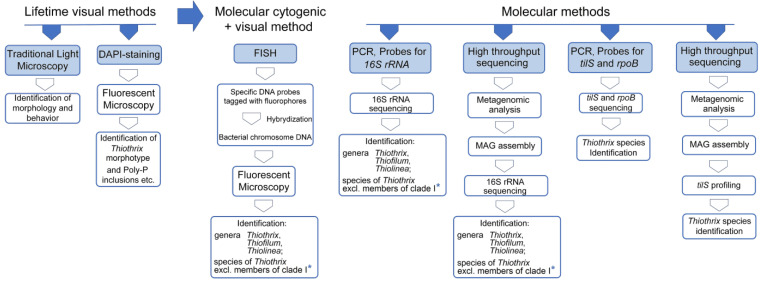
Progress of methods for identification of representatives of *Thiothrix* morphotype from time perspective: light microscopy morphotype identification [6,7]; DAPI staining to confirm bacterial morphotype [71] and identify Poly-P inclusion [47]; FISH for identification of *Thiothrix, Thiofilum* and *Thiolinea* genera and some *Thiothrix* species [31,72,73]; PCR using *tilS* and *rpoB* probes [19] and high-throughput sequencing with *tilS* profiling [19] for *Thiothrix* species identification. The capabilities of the FISH method and the PCR method when using probes for 16SRNA in both methods are current at the time of writing the review and are indicated for the taxonomic groups of *Thiothrix*, *Thiofilum* and *Thiolinea* revised in 2018 [10], with no consideration of the taxonomic status of these groups before 2018. * Not applicable for species identification of *Thiothrix* Clade I, the composition of which is listed in Figure 1, at the date of the review’s preparation.

The significant contribution of the *Thiothrix* morphotype bacteria to the operation of wastewater treatment plants has long been known, leading to the development of methods for their identification using light microscopy (Figure 2). The application of these methods, along with detailed morphological characterization, led to the identification of filamentous bacteria in wastewater treatment plants that were assigned to the genus *Thiothrix* and described as Type 021N, *Thiothrix* I and *Thiothrix* II [6,7]. Therefore, these names were retained in many subsequent articles written by non-taxonomists dealing with microorganisms in wastewater treatment plants [13,14,15,16,17,18]. The original classification of these bacteria outside of the genus *Thiothrix* was further confirmed by Boden and Scott [10], who proposed placing *Thiothrix flexilis* in the genus *Thiofilum* and *Thiothrix disciformis* and *Thiothrix eikelboomii* in the genus *Thiolinea*.

To monitor the population dynamics of *Thiothrix* in wastewater treatment plants, species-specific fluorescence in situ hybridization (FISH) probes are often used, in addition to light microscopy [13,18,26,36,74,75,76,77]. Current phylogenetic data suggest that some of the probes used could also be suitable for representatives of the genera *Thiolinea* and *Thiofilum* [18,75,77].

Since the phylogenetic heterogeneity of *Thiothrix* was long known, sets of different probes were developed to identify all known species of the genus known at that time. Their use showed that mixed populations of the *Thiothrix* morphotype are often found in wastewater treatment plants, and bacteria now assigned to the genus *Thiolinea* can also be found in these mixtures [31].

Quantitative PCR has been used less frequently to study *Thiothrix* populations. For Type 021N, primers were developed and used in combination with Sybr Green to quantify these bacteria in laboratory tests [78]. Genus-specific primers were developed for *Thiothrix* 16S rRNA, which showed that, in the wastewater studied, the largest number of bacteria similar in morphotype to *Thiothrix* belonged to the species *Thiothrix eikelboomii* (= *Thiolinea eikelboomii*) [14]. Asvapathanagul et al. also showed that the ratio of *Thiolinea eikelboomii* to the total number of bacteria (TH/TB) (%) was a better predictor of the secondary effluent volume and quality than the *Thiolinea eikelboomii* abundance alone [14].

However, the rapid development of high-throughput sequencing technologies in recent years has led to the increasingly frequent detection of *Thiothrix* by community profiling against 16S rRNA genes [24,52,79,80]. Moreover, representatives of the genera *Thiothrix* [56,81] and *Thiolinea* [11] were identified in activated sludge by metagenomic analysis.

Although it is known that a mixture of several *Thiothrix* species can occur simultaneously in wastewater treatment plants, the identification of these species has so far been a challenge. The reason for this is that the 16S rRNA phylogenetic marker most commonly used in high-throughput sequencing offers an insufficient resolution to unambiguously assign *Thiothrix* to a particular species. In this regard, to identify *Thiothrix* to the species level, primers for other phylogenetic markers—*tilS* and *rpoB*—were constructed [19]. However, they have not yet been used in the analysis of the microbial communities of wastewater treatment plants, but they have shown good results in the analysis of microbial communities from natural biotopes.

## 4. Metabolic Processes in Wastewater Treatment Involving *Thiothrix* and Prospects for *Thiothrix*’s Further Application

### 4.1. Sulfide Removal

As prominent representatives of the group of sulfur-oxidizing bacteria, members of the *Thiothrix* morphotype have the metabolic potential to remove toxic sulfide from wastewater. The incoming raw sewage, which contains sulfide resulting from the degradation of protein substrates, is the most probable source of *Thiothrix* found in activated sludge from wastewater treatment plants. For example, the same *Thiothrix* phylotype was detected both in influent raw sewage and in activated sludge from Moscow wastewater treatment plants, and its percentage in the influent microbial community was much higher than in activated sludge and treated water (0.79% vs. 0.05% vs. 0.17%); for the list of 16S rRNA gene reads, see Table S1, SM in Ref. [82].

**Figure 3:**
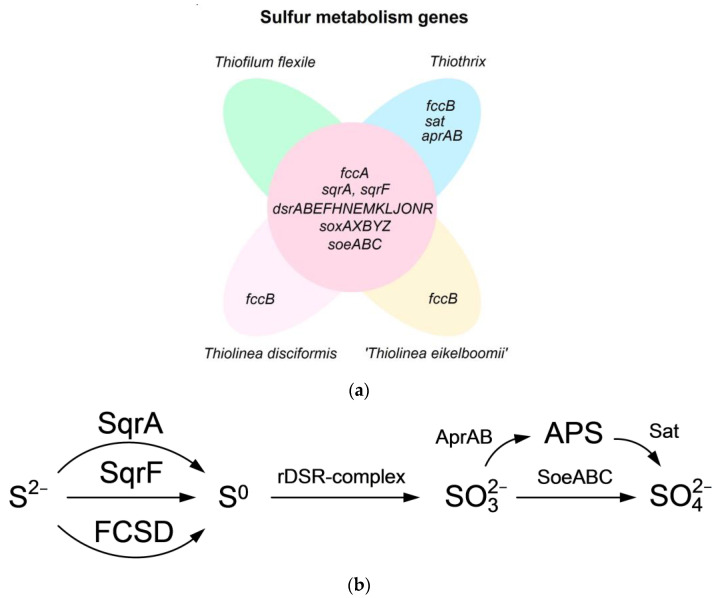
Sulfur metabolism in representatives of *Thiothrix* morphotype. (**a**) EVenn diagram presenting sulfur metabolism genes found in the genomes of the genera *Thiothrix, Thiophilum* and *Thiolinea.* Panel with *Thiothrix* genes is based on pangenome analysis involving 15 genomes of *Thiothrix litoralis* AS^T^, *Thiothrix lacustris* BL^T^, *Thiothrix fructosivorans* Q^T^, ‘*Ca.* Thiothrix putei’, *Thiothrix caldifontis* G1^T^, *Thiothrix winogradskyi* CT3^T^, ‘*Ca.* Thiocaldithrix sulfatifontis’, *Thiothrix subterranea* Ku-5^T^, *Thiothrix* sp. STA_22, *Thiothrix unzii* A1^T^, ‘*Ca*. Thiothrix anitrata’ A52, ‘*Ca.* Thiothrix moscovensis’ RT, *Thiothrix* sp. 207, *Thiothrix nivea* JP2^T^ and ‘*Ca.* Thiothrix singaporensis’ SSD2 and on new 16th genome of ‘*Ca.* Thiothrix namsaraevi’. The genes in the other three panels are shown based on representative species: *Thiolinea disciformis* DSM 14473^T^ and ‘T*hiolinea eikelboomii’* in the case of the genus *Thiolinea*; *Thiofilum flexile* in the case of the genus *Thiofilum.* Figure was prepared using EVenn diagram software (http://www.ehbio.com/test/venn/, accessed on 11 July 2024) [83] and considers data in Refs. [4,5,84]. (**b**) Reactions of sulfur dissimilatory metabolism.

A well-developed system for sulfur metabolism underlies the broad capabilities of representatives of the genus *Thiothrix* to participate in wastewater treatment from sulfur compounds. Genome studies of all known *Thiothrix* species indicate that all of them contain genes for dissimilatory sulfur metabolism (Figure 3a).

The pangenome of the genus *Thiothrix* comprises *sqr* (*sqrF*, *sqrA*) genes encoding sulfide: quinone oxidoreductase, which is involved in the oxidation of hydrogen sulfide to elemental sulfur; *fccAB* genes encoding sulfide-flavocytochrome-*c* dehydrogenase (FCSD); *soxAXBYZ* genes encoding a branched SOX system for the oxidation of thiosulfate to sulfur and sulfate; genes *dsrABEFHNEMKLJONR* encoding the rDSR complex that carries out the oxidation of sulfur to sulfite; *soeABC* genes encoding quinone-dependent sulfite dehydrogenase catalyzing the direct oxidation of sulfite to sulfate; genes *sat* encoding ATP-sulfurylase of the dissimilatory type; and *aprAB* genes encoding APS reductase for indirect sulfite oxidation [5]. A genome analysis of representatives of other *Thiotrichaceae* genera, namely *Thiolinea disciformis*, *Thiolinea eikelboomii* and *Thiofilum flexile*, showed the presence of all genes of dissimilatory sulfur metabolism with the exception of ATP-sulfurylase of the dissimilatory type (*sat*) and APS-reductase (*aprAB*) for indirect sulfite oxidation, as well as the lack of *fccB* genes of the FCSD complex in *Thiofilum flexile* [5].

Thus, among the representatives of the *Thiothrix* morphotype, only representatives of the genus *Thiothrix* have the most developed system of oxidative dissimilatory sulfur metabolism (Figure 3b). This suggests that they are the most universal candidates for systems of water treatment from sulfur-containing compounds.

Based on their morphology, microorganisms similar to *Thiothrix* sp. were identified in the sludge beds of upflow anaerobic sludge blanket (UASB) reactors, where the ability of these organisms to oxidize sulfide was demonstrated [45].

When the sulfide concentrations temporarily increased in a fluidized bed reactor used to remove nitrates from a recirculating marine fish culture system, whitish tufts identified as the *Thiothrix* morphotype rapidly developed [85]. In these systems, representatives of the *Thiothrix* morphotype participated in sulfide oxidation using nitrate or oxygen as an electron acceptor [46]. Bacteria similar in morphotype to *Thiothrix* were also found as part of a composite microbial biofilm for the treatment of sulfide-containing wastewater [70] and in a laboratory biodrainage filter treating high concentrations of hydrogen sulfide [74,86]. Moreover, in the latter case, among the nearly full-length 16S rRNA gene sequences of the entire bacterial community, sequences related to *Thiothrix lacustris* accounted for 38% [86].

The ability of *Thiothrix* to oxidize hydrogen sulfide could explain that enrichment by *Thiothrix* improved the removal of sulfamethoxazole (SMX) (sulfur-containing organic matter) and mitigated SMX-induced stress to other microorganisms [40].

In addition to the biological removal of sulfide, the *Thiothrix* morphotype can be involved in other metabolic processes that proceed during the biological treatment of wastewater. *Thiothrix* was shown to be a significant part of the microbial community involved in the processes of nitrogen and phosphorus removal (1.51%), nitrification–denitrification (9.41%) and the aerobic removal of carbon (4.29%) [87].

### 4.2. Enhanced Biological Removal of Phosphorus

Phosphorus is a key element responsible for the eutrophication of the aquatic environment, which leads to a shift in the equilibrium in the hydroecosystem, subsequently resulting in the development of cyanobacteria, a sharp decrease in the concentration of dissolved oxygen in water, the accumulation of biogenic toxins, the deterioration of water quality, the death of fish and other aquatic organisms and the waterlogging of the water body. In this regard, the removal of phosphorus from wastewater is an important task in preventing the harmful effects of eutrophication in the ecological, economic and social spheres. A biological approach could be among the methods to address this problem. One of the most successful directions of biotechnology is the use of colorless sulfur bacteria of the genus *Thiothrix* in “enhanced biological phosphorus removal” (EBPR) systems. Based on the data of a pangenomic analysis, it can be assumed that the members of the genus *Thiothrix* can behave as phosphorus-accumulating organisms with mixotrophic metabolism during phosphorus removal and, at the same time, use the oxidation of the intracellular sulfur pool as an additional energy source along with poly-β-hydroxy-alkanoates (PHA). A large number of such systems are currently operating successfully around the world. The idea of using biologically safe methods for phosphorus removal based on modified activated sludge systems was implemented in the late 1950s when the “enhanced biological phosphorus removal” (EBPR) system was developed [88]. In an EBPR treatment system, phosphate-accumulating organisms (PAOs) accumulate increased amounts of polyphosphates in their cells and thus biologically remove phosphorus from wastewater [89].

**Figure 4:**
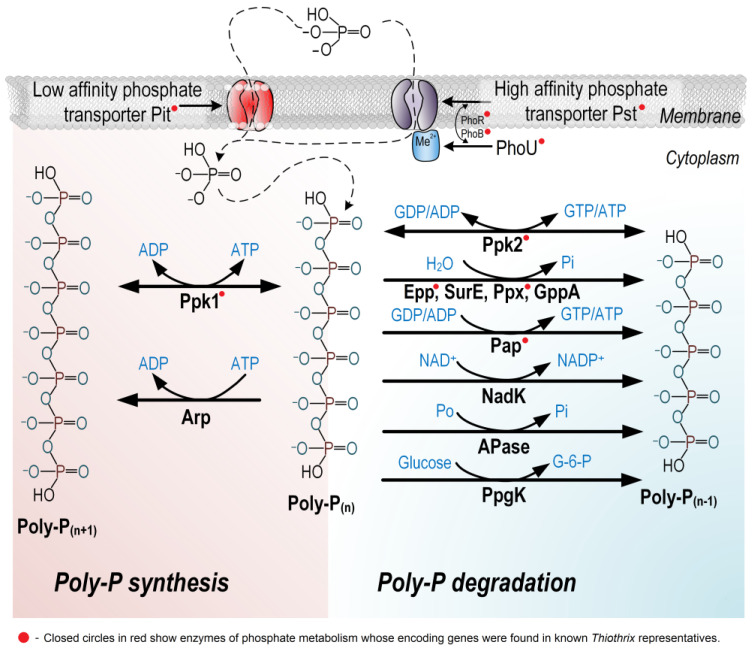
Possible pathways of phosphorus metabolism in bacterial cells. For the depicted names of the enzyme proteins, see the text. The figure was adapted from Roy et al. [92] and Gardner et al. [93] under the terms of the Creative Commons Attribution 4.0 International License. *Thiothrix* enzymes are shown based on the published data [4,11,47,49,56,84,94].

A large number of enzyme proteins are known to be involved in the biological cycle of phosphorus (Figure 4). In bacteria, the fully characterized enzymes that enable the reversible accumulation of phosphorus in the form of polyphosphate (poly-P) are polyphosphate kinases Ppk1 (coupled to ATP dephosphorylation) [90] and Ppk2 (coupled to ATP/GTP dephosphorylation) [91], encoded by the *ppk1* and *ppk2* genes, respectively. It is quite likely that, in addition to this enzyme, there are still unknown mechanisms for poly-P synthesis, such as the Arp complex (*arpABCDEFGH*), which catalyzes poly-P synthesis in archaea and was also shown to have distant homologs in bacteria. Bacterial enzymes that regulate poly-P degradation include exopolyphosphatases Ppx, Epp, GppA (*ppx, epp* and *gppA*); 50/30-nucleotidase SurE (*surE*); poly-AMP phosphotransferase PAP (*pap*); NAD kinase NadK (*nadK*); alkaline phosphatases APases (e.g., *phoX*, *phoD*, *phoA*); and polyphosphate-dependent glucokinase PpgK (*ppgK*). In addition to these enzymes, P_i_-transporter Pit, encoded by the *pit* gene (having low substrate affinity), and the ABC-type P_i_-transporter Pst, formed by four proteins encoded by the *pstSCAB* genes (having high substrate affinity), are used to transport phosphate molecules into the cell [92]. Closely related to the Pst complex are the two-component signaling proteins PhoR and PhoB (*phoR* and *phoB*) and a cytoplasmic protein PhoU (*phoU*), which regulates Pi entry into the cell under conditions of phosphate deficiency. According to the hypothetical scheme proposed by Gardner et al. [93], the metal-binding protein PhoU performs a negative signaling function, providing the bacterial cell with a mechanism to sense the level of P_i_ in the environment.

A successfully operating EBPR system requires that a mixed microbial consortium (MMC) be exposed to cyclic anaerobic–aerobic conditions while simultaneously receiving sufficient volatile fatty acids (VFAs) so as to stimulate the metabolism necessary for EBPR activation. Due to this mode of operation, MMC is enriched in phosphorus-accumulating organisms (PAOs).

The physiological group of PAOs comprises representatives of various lines, including ‘*Ca.* Accumulibacter’, *Tetrasphaera* (later ‘*Ca.* Phosphoribacter’ [95]), *Microlunatus phovovorus*, *Dechromonas*, *Thiothrix* and others [96]. ‘*Ca.* Accumulibacter’ and ‘*Ca.* Phosphoribacter’ are considered the main ones [97].

**Figure 5:**
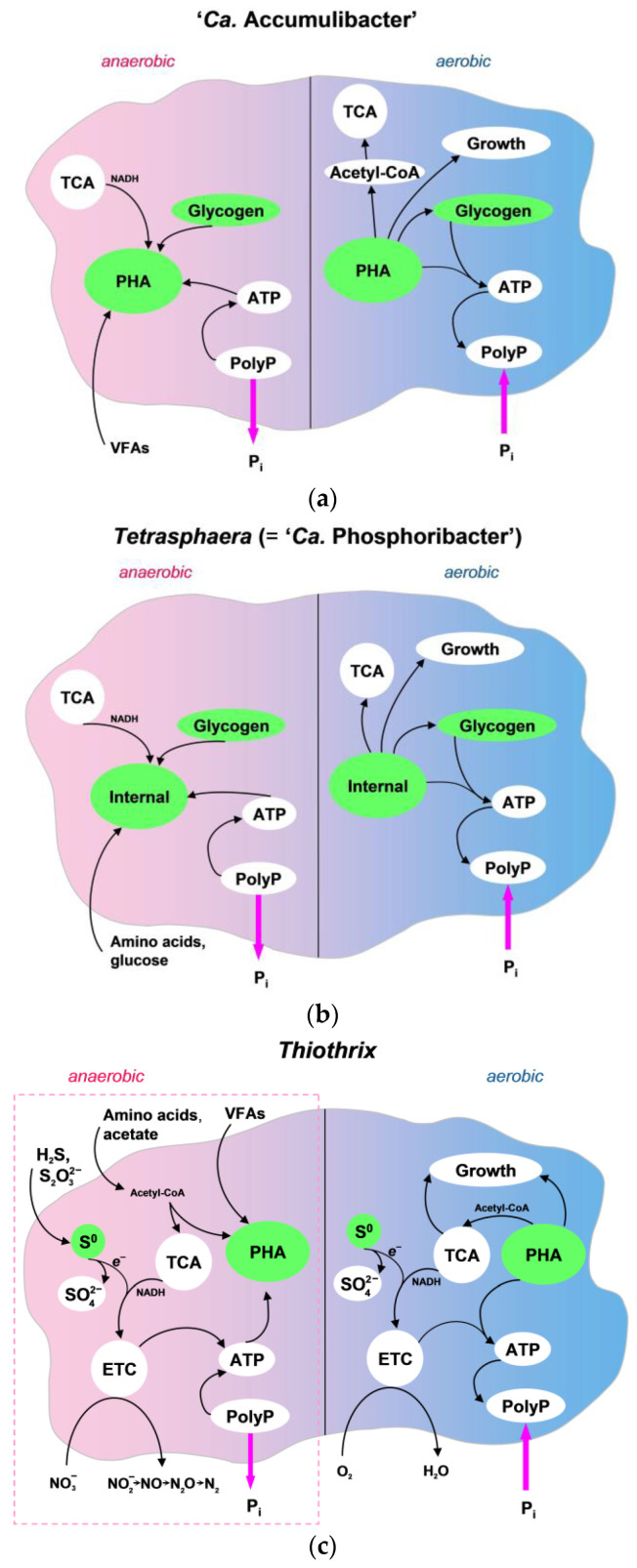
PAO metabolism in (**a**) ‘*Ca.* Accumulibacter’ and (**b**) *Tetrasphaera* (= ‘*Ca.* Phosphoribacter’ [96]) and (**c**) predicted functional potential members of the genus *Thiothrix*. The schemes in panels (**a**,**b**) are based on Nielsen et al. [97] under the terms of the Creative Commons Attribution 4.0 International License; panel (**c**) considers data from published articles [4,5,47,56,94]. In panel (**c**), the metabolic pathways outlined by the dashed line are putative. Internal, internal reserve substances in the cell; PHA, poly-β-hydroxy-alkanoates; S^0^, elemental sulfur; VFAs, volatile fatty acids; PolyP, polyphosphate; TCA, tricarboxylic acid cycle; ETC, electron transport chain.

PAO metabolism by ‘*Ca.* Accumulibacter’ is considered classical. These bacteria, under anaerobic conditions, (i) take up VFAs and store them in the form of the intracellular polymer PHA, (ii) use glycogen as a source of reducing equivalents (carbon is also included in PHA) and (iii) hydrolyze internal polyphosphate (poly-P) to form the essential ATP. Under aerobic conditions, PAOs (i) utilize the PHA reserves to grow and replenish the glycogen reserves and (ii) use the ATP generated by oxidative phosphorylation to take up and convert phosphate to poly-P [98] (Figure 5a).

Unlike ‘*Ca.* Accumulibacter’, representatives of *Tetrasphaera* spp. do not possess the ability to store PHA. However, like representatives of ‘*Ca.* Accumulibacter’, they synthesize poly-P inside cells aerobically and use it anaerobically. It is assumed that amino acids and glucose are substrates in the cycle of poly-P transformation in anaerobiosis. During the reaction, these substrates are presumably converted into free amino acids and glycogen, although the role of glycogen is unclear, since it is not found in the activated sludge of *Tetrasphaera* spp. representatives [95] (Figure 5b).

It was shown that members of the genera *Thiothrix* [47] and *Thiolinea* [11] can play a key role in biological phosphorus removal. As shown under aerobic conditions in EBPR systems, in an environment with low organic content but enriched in reduced sulfur compounds, *T. caldifontis* could behave as an efficient phosphate-removing PAO with mixotrophic metabolism. Phosphorus removal from the environment in the aerobic phase proceeds with the oxidation of the intracellular sulfur pool to provide PAO with an additional source of energy [47]. In the same EBPR system, under anaerobic conditions, this species can store carbon in the form of PHA and generate the required energy via the hydrolysis of poly-P stored in the aerobic phase. In the aerobic period, PHA was used as the source of carbon and energy for the growth and synthesis of poly-P (Figure 5c). On the other hand, *Thiothrix* sp. that received glutamate as the only source of nitrogen and carbon was able to store poly-P, probably avoiding PHA synthesis [99].

It should be noted that the growth conditions in EBPR systems differ markedly from those of batch cultivation and often provide preferences for new and rare species. Several representatives of *Thiothrix* were described resulting from the assembly of MAGs obtained from the activated sludge in EBPR systems [11,56,81]. One of the resulting MAGs was closely related to the species *Thiothrix disciformis* (= *Thiolinea disciformis*) [10,11] and two MAGs were described as new species with *Candidatus* status, namely ‘*Ca.* Thiothrix moscovensis’ and ‘*Ca.* Thiothrix singaporensis’ [56].

**Figure 6:**
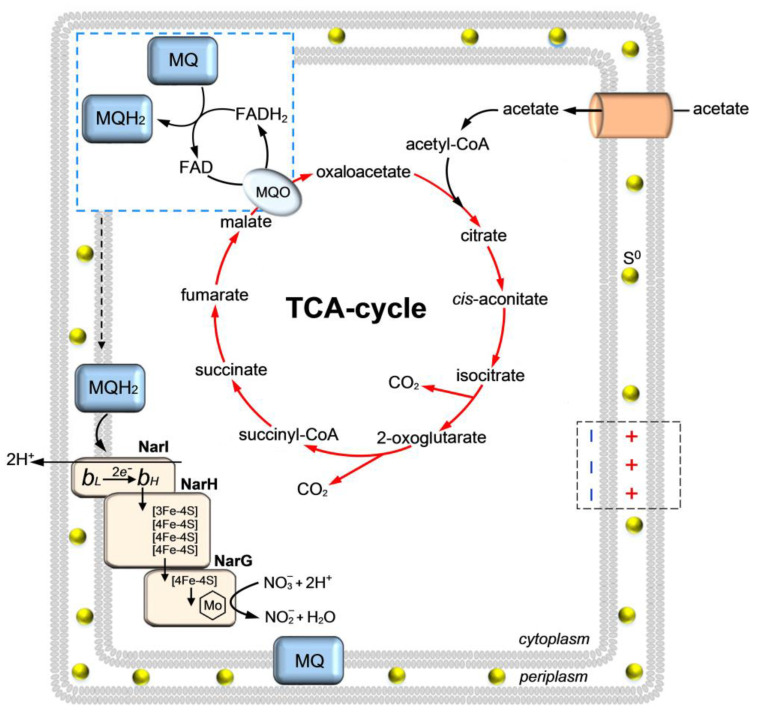
The role of malate: quinone oxidoreductase (MQO) in *Thiothrix* metabolism as determined from experimental and genomic data. MQ and MQH_2_—oxidized and reduced forms of menaquinone, respectively; NarI, NarH and NarG—dissimilatory nitrate reductase (Nar) subunits with a cytoplasmically oriented active center; *b_L_* and *b_H_—*low-potential and high-potential cytochrome *b* in the membrane-spanning NarI subunit; [3Fe-4S] and [4Fe-4S]—redox-active iron-sulfur clusters; arrows within Nar subunits indicate the electron pathway between the redox groups. Insets: in the black dashed line on the right side of the figure—the direction of electrical potential at the cytoplasmic membrane, which is charged negatively at the cytoplasmic side and positively at the periplasmic side during the process of nitrogen respiration; in the blue dashed line in the upper left part of the figure—reactions occurring inside the cytoplasmic membrane. Figure is based on Mardanov et al. [56]; Trubitsyn et al. [105]; Guigliarelli et al. [111]; and Martinez-Espinosa et al. [106] under the terms of the Creative Commons Attribution 4.0 International License.

An important factor determining the function of the representatives of the genus *Thiothrix* as a PAO is the carbon source and the capacity for anaerobic respiration. Due to a rare metabolic feature of ‘*Ca.* Thiothrix moscovensis’ and ‘*Ca.* Thiothrix singaporensis’, namely the presence of FAD-dependent malate: quinone oxidoreductase (MQO) instead of NAD-dependent malate dehydrogenase (MDH), these two species can effectively grow and function as PAOs in EBPR under organotrophic conditions, using acetate as a substrate (Figure 6) [56]. The same metabolic potential is inherent in all representatives of the genera *Thiothrix*, *Thiolinea* and ‘*Ca*. Thiocaldithrix’ since their genomes contain the *mqo* gene instead of the *mdh* gene [4,5]. Presumably, these members of *Thiothrichaceae*, except for species lacking nitrate reductase genes, are better able to overcome the anaerobic phase in the EBPR cycle in the presence of nitrate, allowing them to activate nitrate-mediated respiration (Figure 6). This may be facilitated by the following factors, which, under certain unfavorable conditions, can presumably create advantages for species with MQO instead of MDH or (MDH+MQO), despite the fact that, formally, these two enzymes catalyze the same reaction.

1. MQO is selectively activated 3.4–4-fold during microbial growth on acetate compared to growth on glucose, whereas the corresponding figure for MDH is 1.8 [100,101].

2. In the case of MQO catalysis, but not MDH catalysis, there is no dependence of the reaction progression malate → oxaloacetate on the NAD/NADH balance, because the cofactor of MQO is the FAD/FADH pair, while NAD/NADH is involved in the MDH-catalyzed reaction.

3. Moreover, in the case of MQO catalysis, there is no dependence of the reaction on the malate/oxaloacetate balance because the reaction is exergonic (Δ*G°′* = −18.9 and −55.0 kJ/mol when the electron acceptor is MQ or UQ, respectively) and proceeds spontaneously. In contrast, the reaction with MDH is endergonic (Δ*G°′* = +28.6 kJ/mol) and does not proceed in the forward direction under normal conditions according to the laws of chemistry.

4. It follows from (3) that the simultaneously use of MQO and MDH is disadvantageous under conditions of an energy deficit and a lack of oxygen, because they lead to a futile cycle, where oxaloacetate, reduced from malate by MQO, is converted to malate again due to the reverse reaction catalyzed by MDH. The end result of such a reaction is equivalent to the reaction of noncoupled NADH: quinone oxidoreductase type II (common in *Bacillus* species and many other bacteria), i.e., the production of reduced quinone, but at a higher cost than in the MQO-catalyzed reaction.

5. One possible way to avoid this wastefulness and not waste an “expensive” reducing agent on an unprofitable reaction (see (4)) might be to either block the available MDH or replace MDH with MQO. Switching to MQO catalysis preserves oxaloacetate as an essential substrate for citrate synthase in TCA, as well as for gluconeogenesis and the synthesis of aspartate branch amino acids.

6. The outcome of switching individual microorganisms to MQO maintenance may be the more reliable functioning of TCA under some unfavorable conditions, namely anaerobiosis (respiration with less favorable oxidants compared to oxygen, e.g., nitrate), under carbon-containing substrate limitations, and others.

The electron acceptor from MQO can be menaquinone, which has a more negative redox potential (*E′*_0_ = −0.075 V) compared to ubiquinone (*E′*_0_ = +0.100 V) and is almost ideally matched to the FAD/FADH pair (*E′*_0_ = −0.060 V) (cofactor in MQO) to form a unit in the electron transport chain. It is worth noting that, in contrast to strictly aerobic and strictly anaerobic bacteria, both ubiquinones and menaquinones are synthesized in membranes in facultatively anaerobic bacteria, which include representatives of *Thiothrix*. As shown in *E. coli*, the synthesis of all types of menaquinones increases under anaerobic conditions and exceeds the total amount of ubiquinone three-fold [102,103], which makes menaquinone a more likely electron acceptor from MQO under anaerobic conditions.

Another notable change in the profile of biosynthesis products induced by anaerobiosis in the six *Thiothrix* species studied is a 2.4–6.5-fold increase in the expression of the *narG* gene [104,105] encoding the catalytic subunit of the dissimilatory nitrate reductase NarGHI anchored in the cytoplasmic membrane. It has been shown that the induction of expression by anaerobiosis is accompanied by an increase in the total activity of nitrate reductase in the cells.

Experimental observations on different bacterial species possessing MQO demonstrate good growth rates on acetate and organics at high MQO activity and a significant decrease in growth in MQO deletion mutants in *Corynebacterium glutamicum* [106] and *Pseudomonas aeruginosa* [107], slow growth on fermentable carbon sources and no growth on non-fermentable carbon sources in *Micobacterium stegmatis* [108]. The deletion of *mqo* in *Mycobacterium tuberculosis* leads to stress and impairs the survival of the bacterium [109]; in *Pseudomonas fluorescens*, disruptions in the *mqo* gene led to the loss of the ability to colonize on the root system of tomato [110].

All of the above facts point to an important energetic role of MQO in coupling with nitrate respiration. Although the respiratory chain with Nar enzyme complex as a terminal oxidase and nitrate as an electron acceptor are characterized by lower energetic efficiency as compared to oxygen respiration, the energetic capacity of respiration involving nitrate is comparable to that of the aerobic respiration process (nitrate respiration allows bacteria to transform the energy of substrates into universal energy form with electric potential on the membrane, comparable in magnitude to oxygen respiration). The change in free energy during the oxidation of one molecule of glucose by molecular oxygen (Δ*G°′* = −2870 kJ/mol) is of the same order as the oxidation of the same substrate under anaerobic conditions by nitrate reduced to nitrite (Δ*G°′* = −1770 kJ/mol). As shown in a pangenomic study, almost all members of the genus *Thiothrix,* with the exception of ‘*Ca*. Thiothrix anitrata’ A52 and ‘*Ca*. Thiothrix sulfatifontis’ KT, possess the capacity for anaerobic respiration using nitrogen-containing compounds [94], which indicates the high potential of *Thiothrix* representatives to persist in bioreactors in the anaerobic phase.

According to Rey-Martínez et al., during the long-term operation of the EBPR system with glutamate as the sole source of carbon and nitrogen, the content of members of the genus *Thiothrix* could be 37%, with *Thiothrix* members able to participate in phosphorus removal by accumulating poly-P [99]. However, another study showed that although *Thiothrix* was one of the dominant PAOs at different stages of EBPR bioreactor operation, it was more abundant with propionic acid as the carbon source than when glutamate was used [112]. The use of glycine as a carbon source had an adverse effect on *Thiothrix* and other PAOs in the EBPR system [96].

It should be noted that the explosive growth of members of the genus *Thiothrix* leading to unfavorable sludge bulking can be observed in EBPR systems under changes in cultivation conditions, such as stopping the use of the nitrification inhibitor allylthiourea, which is used to stimulate the growth of *Tetrasphaera*-related PAOs [113].

The intensive study of *Thiothrix* metagenomes in the last decade has revealed the significant potential of the species of this genus to address the challenge of phosphate removal from the environment. In the two genomes of ‘*Ca.* Thiothrix moscovensis’ and ‘*Ca.* Thiothrix singaporensis’ derived from laboratory EBPR, as well as in the genomes of *T. lacustris* BL^T^, *T. caldifontis* G1^T^ and *T. nivea* JP2^T^, the genes *phoURB* and *pstSACB*, encoding the systems of signaling regulation and the transport of inorganic phosphate into the cell, and the genes *ppk1* and *epp*, whose products catalyze poly-P synthesis and degradation, were identified [56]. Rubio-Rincón et al. documented the visual mass accumulation of poly-P inclusion granules in the cells of *T. caldifontis*, corroborated by data on the presence of gene *ppk2* in its genome [47]. To date, we have detected the presence of multiple genes *ppk2* in the available genomes of the most identified species of the genus *Thiothrix* (in the following, access to only one of several genes in each species is shown), namely *T. unzii* A1^T^ [QTR55213.1] [94], *T. fructosivorans* Q^T^ [QTX11709.1] [94], *Thiothrix subterranea* Ku-5^T^ [WML89006.1] [19], ‘*Ca.* Thiothrix putei’ [WGZ96527.1] [5], ‘*Ca.* Thiothrix singaporensis’ [QLQ34282.1] [114], *T. lacustris* BL^T^ [WML90248.1] [19], *T. nivea* JP2^T^ [EIJ34895.1], *T. litoralis* AS^T^ [QTR45363.1] [94], ‘*Ca*. Thiothrix anitrata’ A52 [QTR51311.1] [94], ‘*Ca.* Thiocaldithrix sulfatifontis’ [UOG93965.1] [55], *T. winogradskyi* CT3^T^ [UJS26581.1] [55], ‘*Ca.* Thiothrix sp. Deng01’ [WP_324693012.1] [115] and a number of unidentified *Thiothrix* species, as well as the members of the other *Thiotrichaceae* genera, ‘*Ca.* Thiocaldithrix dubininis’ [WGZ92350.1] [5], *Thiofilum flexile* DSM 14,609 (formerly *Thiothrix flexilis*) [ARCL01000001.1] and *Thiolinea eikelboomii* (formerly *Thiothrix eikelboomii*) [SKA88474.1]. According to Matsuura et al., *Thiothrix disciformis* (= *Thiolinea disciformis*) possesses genes for the major systems of phosphate metabolism (*ppk*, *ppx*, *pst* and *pit*), which include two different phosphate transport systems, Pst and Pit, with high and low affinity for phosphate [11]. A metagenomic analysis performed by the research group of Dong et al. also showed that the identified representatives of the genus *Thiothrix* have the genes of phosphate transport, *pit* and *phnCDE* and other genes of phosphate metabolism: *phoURB*, *pst*, *ppk* [49]. Most recently, Chernitsyna et al. reported that the genes encoding the systems of Pi transport into the cell (*pstDCABS*) and the signaling regulation of phosphate transport (*phoURB*), as well as the systems of poly-P synthesis and degradation, namely polyphosphate kinase (*ppk1*) and exopolyphosphatase (*ppx*), were found in the MAG-assembled genome of ‘*Ca.* Thiothrix namsaraevi’, a new member of the genus *Thiothrix* from Lake Baikal [84]. Ravin et al. identified genes pstDCABS, phoURB and *epp*, as well as genes *ppk1* and *pap*, for poly-P synthesis and hydrolysis in three species of *Thiothrix*, *T. subterranea* sp. nov. Ku-5, *T. litoralis* sp. nov. AS and ‘*Ca.* Thiothrix anitrata’ sp. nov. A52, obtained from other geographical locations in Russia—a coal mine, the White Sea littoral and a sulfur spring in the Volgograd region [94]. According to a summarized pangenomic analysis, the core genome of 12 *Thiothrix* species (*T. winogradskyi* CT3^T^, *T. lacustris* BL^T^, *T. litoralis* AS^T^, *T. subterranea* Ku-5^T^, *T. caldifontis* G1^T^, *T. unzii* A1^T^, *T. nivea* JP2^T^, *T. fructosivorans* Q^T^, ‘*Ca.* Thiothrix moscovensis’ RT, ‘*Ca*. Thiothrix anitrata’, ‘*Ca*. Thiothrix sulfatifontis’ KT, MAG of *Thiothrix* sp. 207) contains genes for the main systems of phosphate metabolism, with the exception of only one of the three genes of the cellular phosphate transport system, *pstB*, which may be absent in some species [4]. As can be seen from the recent publications, almost all representatives of the genus *Thiothrix*, with a few exceptions, have genes for the signal regulation of Pi import into the cell, namely *phoUR* or *phoURB*, encoding a system of two or three corresponding proteins PhoU, PhoR and PhoB, as well as genes of Pi transporters, *pstDCABS*, *pit* and *phnCDE*; polyphosphate degradation and synthesis, *ppk1* and *ppk2*; exopolyphosphatases, *epp* and *ppx*; and poly-AMP phosphotransferase, *pap* (Figure 4).

### 4.3. Nitrogen Removal

In addition to phosphorus, nitrogen is one of the two major biogenic elements that threaten to upset the equilibrium in water bodies under anthropogenic impacts. While phosphorus and especially phosphates, which pose the greatest problem for water treatment, are mainly responsible for ecological damage to natural inland water reservoirs, nitrogenous compounds are more problematic for marine water reservoirs, although both types of compounds are dangerous for any water body under uncontrolled inputs. Members of the *Thiothrix* morphotype are prominent representatives of sulfur-oxidizing bacteria with metabolic potential to remove toxic nitrates from wastewater. For many species of the genus *Thiothrix*, the ability to denitrify during mixotrophic growth in the presence of reduced sulfur and organic compounds (lactate and acetate) has been experimentally demonstrated [105]. No data are available on the ability of *Thiothrix* pure cultures to denitrify in the absence of reduced sulfur compounds. An analysis of the pangenome of the genus *Thiothrix* showed the presence of denitrification genes in the variable part of the pangenome. All members of the genus, with the exception of ‘*Ca.* Thiothrix anitrata’ and ‘*Ca*. Thiothrix sulfatifontis’ KT, have genes encoding at least one form of nitrate reductase, which reduces nitrate to nitrite (Table 1). Most strains contain genes for nitrite reductase (*nirS*), which reduces nitrite to nitric oxide, and nitric oxide reductase NorBC, which reduces nitric oxide to nitrous oxide. The nitrous oxide reductase NosZ genes were found in only one of the strains involved in the pangenomic analysis [4].

**Figure 7:**
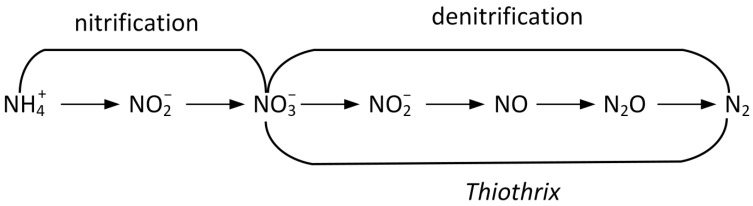
Nitrification–denitrification reactions. In the processes of the transformation of nitrogenous compounds leading to nitrogen removal from wastewater, representatives of the genus *Thiothrix* are able to perform the denitrification step.

In case of uncontrolled inputs due to effluents from animal farms, agricultural factories, industrial companies and the sewage systems of residential complexes, high concentrations of nitrogenous compounds lead to the destructive eutrophication of water bodies and ammonium toxicity to fish and other aquatic fauna as well as flora. Kosgey et al. presented traditional schemes for the biological treatment of wastewater contaminated with nitrogenous compounds [116]. These schemes are based on the use of specialized bacteria with different types of nitrogen metabolism, which allows the sequential conversion of toxic ammonium ions (NH_4_^+^) to safe dinitrogen molecules (N_2_) through a chain consisting of nitrifier bacteria, which oxidize ammonium (AOB) and nitrite (NOB), and denitrifier bacteria, which reduce nitrate at the expense of organic carbon, which acts as an electron donor (Figure 7). In this scheme, representatives of the *Thiothrix* morphotype can play a role in the last stage.

Representatives of the *Thiothrix* morphotype are often found in nitrification–denitrification systems [32,117,118,119,120]. Their role in these systems can involve denitrification coupled with the oxidation of reduced sulfur compounds. In this case, sulfides can be used as an electron donor for the reduction of nitrates and nitrites [121]. There are reports that the *Thiothrix* morphotype is capable of nitrate-mediated sulfide oxidation [46]. It was shown that bacteria similar in morphotype to *Thiothrix* began to play the role of denitrifiers in the simultaneous nitrification–denitrification system when the oxygen concentration decreased [122].

Zhao et al. found a large number of bacteria of the *Thiothrix* morphotype in biofilm reactors with simultaneous nitrification–denitrification processes, where pomelo peel, which can biodegrade to form sulfide, served as a carrier [123]. This explains the appearance of dominant representatives of the *Thiothrix* morphotype in the system and the presence of denitrification associated with the oxidation of reduced sulfur compounds.

### 4.4. Denitrifying Removal of Phosphorus

Two methods of phosphorus removal by denitrifying have been described. One is based on the operation of both denitrification and phosphorus accumulation reactions in the same microorganism. Another variant is based on the separate running of these reactions, where denitrification is carried out by one microorganism and phosphorus accumulation by another [124]. Denitrifying organisms that accumulate phosphate can simultaneously remove nitrogen and phosphorus under rotational anaerobic and anoxic/aerobic conditions [125].

The role of bacteria of the *Thiothrix* morphotype in denitrifying phosphorus removal has been suggested in different studies. The presence of these bacteria in the denitrifying phosphorus removal system was mentioned, but their function as denitrifying or dephosphorizing bacteria was not considered [126]. Other studies indicate a dephosphorizing role for the bacteria of the *Thiothrix* morphotype in such systems. For example, Li et al. found that bacteria similar in morphotype to *Thiothrix* played a crucial role in the accumulation of precipitated phosphate in a reactor with simultaneous nitrification–denitrification and phosphorus removal [127]. Moreover, Ai et al. stated that, in a wastewater treatment system where denitrifying phosphorus removal served as the primary pathway of phosphorus removal, the *Thiothrix* morphotype was associated with phosphorus removal [128].

In addition, bacteria similar in morphotype to *Thiothix* are believed to be capable of denitrifying phosphorus removal [48]. An analysis of metagenomes from a biofilm reactor in which denitrifying phosphorus removal was performed showed that bacteria similar in morphotype to *Thiothrix* had genes for denitrification and *pit* genes for the phosphate transporter [49]. Thus, representatives of the *Thiothrix* morphotype are potentially capable of denitrifying phosphorus removal within a single organism.

## 5. Factors Affecting *Thiothrix* Proliferation

In wastewater treatment systems, due to the significant role of bacteria similar in morphotype to *Thiothrix*, it is important to consider factors that can lead to their increased growth and methods to control it.

Bacteria of the *Thiothrix* morphotype are able to dominate in activated sludge in the presence of thiosulfate, which can be related to the ability of these bacteria to grow mixotrophically using reduced sulfur compounds as an energy source [26]. Consistent with this phenomenon, the core genome of the genus *Thiothrix* was found to contain genes encoding sulfur metabolism complexes: the SQR and FCSD complexes, participating in the oxidation of sulfide to polysulfide/sulfur; the Sox complex (*soxAXBYZ*), which participates in the branched pathway of thiosulfate oxidation to sulfur and sulfate; and the rDsr system (*dsrABCEFHEMPKJOL*), which participates in the subsequent oxidation of elemental sulfur [4].

It was shown that the presence of sulfide in wastewater treatment systems can evoke the active growth of the *Thiothrix* morphotype [44,47,85]. High levels of dissolved oxygen (DO) are detrimental to bacteria similar in morphotype to *Thiothrix* and inhibit their growth, while, conversely, low DO values favor the growth of these bacteria [21]. This is due to the fact that they belong to the group of colorless sulfur-oxidizing bacteria, which are known to be catalase-negative and prefer growth in narrow microaerobic eco-niches where fewer toxic reactive oxygen species are produced. At low DO levels and low nitrate concentrations, bacteria of the *Thiothrix* morphotype gain a competitive advantage over other microorganisms due to the ability to accumulate nitrates intracellularly, which leads to the explosive growth of these bacteria and activated sludge bulking [129]. This phenomenon is related to the ability of bacteria of the *Thiothrix* morphotype to utilize nitrate as an alternative electron acceptor. The appearance of bisphenol A in wastewater also leads to an increase in the abundance of the *Thiothrix* morphotype [33].

To suppress the growth of *Thiothrix*, the addition of polyaluminum chloride and a decrease in VFAs were tested, but these approaches were ineffective in resolving the problem conclusively [34]. Although a correlation was observed between the propionic acid content and the Type 021N-mediated bulking of sludge, this relationship was, in reality, an indirect effect of the inhibition of flocculating microorganisms, which gave filamentous bacteria a competitive advantage in growth [29].

Several methods have been demonstrated to reduce the abundance of bacteria similar in morphotype to *Thiothrix* in wastewater. These include the use of intermittent fasting [34], moderate oxygen concentrations with low aerobic periods in the surrounding habitat [130] and the addition of 1‰ hydrogen peroxide [80], the backwashing of membrane reactors with NaClO in wastewater systems [65] and even exposure to a magnetic field [79].

Regarding the growth rate of bacteria currently assigned to the genus *Thiolinea*, it was demonstrated to have a statistically significant correlation with the food/mass ratio (daily activated sludge load, defined as the ratio of the daily amount of incoming organic pollutants F to ash-free substance of activated sludge M (F:M)) and the ammonium ion concentration in the primary effluent, as well as an inverse correlation with the dissolved oxygen concentration in the microaerobic selector [14].

## 6. Conclusions

Thus, in wastewater treatment systems, bacteria of the *Thiothrix* morphotype are more often found in activated sludge or in membrane bioreactors and much more rarely in biofilms. Modifications to the operating parameters of biological treatment systems can lead to the explosive growth of the *Thiothrix* morphotype and result in system disruption. The proliferation of the *Thiothrix* morphotype is enhanced under low concentrations of dissolved oxygen and in the presence of sulfide and bisphenol A and can be suppressed by peroxide, hypochlorite or exposure to a magnetic field. Historically, representatives of this morphotype were detected based on their morphology; later, FISH probes and PCR primers were developed for their detection; and, now, high-throughput sequencing methods are increasingly used for this purpose. *Thiothrix*-like bacteria have a flexible metabolism, which allows them to perform a number of biological wastewater treatment processes, such as sulfide oxidation, denitrification and enhanced biological phosphorus removal. For bacteria belonging to the genus *Thiothrix*, a pangenome has been obtained, which makes it possible to perform the genetic determination of these metabolic processes. Obtaining pangenomes of the genera *Thiofilum* and *Thiolinea* is a task for further research and could lead to a better understanding of the metabolism of these genera and to the synthesis of data on the metabolism of the representatives of the *Thiothrix* morphotype in wastewater treatment systems. Prospects for the further development of wastewater treatment with the participation of representatives of the *Thiothrix* morphotype include the study of the possibility of using representatives of this group in the removal of phosphorus compounds in EBPR, as well as in the treatment of hydrogen sulfide. Namely, due to the knowledge of their metabolic potential, we wish to draw the attention of researchers not only to the possibility of identifying the presence of representatives of the *Thiothrix* morphotype in various wastewater treatment systems, etc., but also to the possibility of the targeted introduction of these organisms in the process of phosphorus and hydrogen sulfide removal.

## Figures and Tables

**Table 1:** Distribution of nitrogen metabolism genes in *Thiothrix* genomes. Genes: *narG*, membrane-bound nitrate reductase; *napAB*, periplasmic nitrate reductase; *nasA*, assimilatory nitrate reductase; *nasB*, assimilatory nitrite reductase; *nirS*, dissimilatory nitrite reductase; *nirBD*, assimilatory nitrite reductase; *norBC*, nitric oxide reductase; *nosZ*, nitric oxide reductase. Table was based on Ravin et al. [5] and Chernitsyna et al. [84] under the terms of the Creative Commons Attribution 4.0 International License.

Strain	Dissimilatory Nitrate Reduction				Assimilatory Nitrate and Nitrite Reduction	
	NO_3_^−^ → NO_2_^−^	NO_2_^−^ → NO	NO → N_2_O	N_2_O → N_2_	NO_3_^−^ → NO_2_^−^	NO_2_^−^ → NH_3_
MAG *Thiothrix* sp. 207	*narGHIJ*, *napAB*	*nirS*	*norBC*	*nosZ*	*nasA*, *nasD*	*nirBD*
MAG *Thiothrix* sp. STA 22	*narGHIJ*	*nirS*	*norBC*	*nosZ*	*nasA*, *nasD*	*nirBD*
*T*. *litoralis* AS^T^	*narGHIJ*	*nirS*	*norBC*	-	*nasA*, *nasD*	*nirBD*
*T*. *fructosivorans* Q^T^	*narGHIJ*	*nirS*	*norBC*	-	*nasA*, *nasD*	*nirBD*
*T*. *caldifontis* G1^T^	*narGHIJ*	*nirS*	*norBC*	-	*nasA*, *nasD*	*nirBD*
*T*. *winogradskyi* CT3^T^	*narGHIJ*	*nirS*	*norBC*	-	*nasA*, *nasD*	*nirBD*
*T*. *unzii* A1^T^	*narGHIJ*	*nirS*	*norBC*	-	*nasA*, *nasD*	*nirBD*
‘*Ca*. Thiothrix singaporensis’ SSD2	*narGHIJ*	*nirS*	*norBC*	-	*nasA*, *nasD*	*nirBD*
‘*Ca*. Thiothrix moscovensis’ RT	*narGHIJ*	-	*norBC*	-	*nasA*, *nasD*	*nirBD*
‘*Ca*. Thiothrix putei’ GK-02	*narGHIJ*	-	*norBC*	-	-	-
*T*. *subterranea* Ku-5^T^	*narGHIJ*	-	-	-	*nasA*, *nasD*	*nirBD*
*T. lacustris* BL^T^	*narGHIJ*	-	-	-	*nasA*, *nasD*	*nirBD*
‘*Ca*. Thiothrix sulfatifontis’ KT	-	-	-	-	*nasA*, *nasD*	*nirBD*
*T*. *nivea* JP2^T^	*napAB*	-	-	-	*nasA*, *nasD*	*nirBD*
‘*Ca*. Thiothrix anitrata’ A52	-	-	-	-	-	-
‘*Ca*. Thiothrix namsaraevi’	*narGHIJ*	-	-	-	*nasA*	*nirBD*

## Data Availability

The original contributions presented in the study are included in the article; further inquiries can be directed to the corresponding authors.
